# Treatment of recurrent pregnancy loss in women with euthyroid-based thyroid peroxidase antibody syndrome

**DOI:** 10.25122/jml-2023-0070

**Published:** 2023-08

**Authors:** Sheimaa Mohsen Mohammad

**Affiliations:** 1 Obstetrics and Gynecology Department, College of Medicine, University of Al-Qadisiyah, Al Diwaniya, Iraq

**Keywords:** miscarriage, recurrent pregnancy loss, thyroid peroxidase antibody, thyroxine

## Abstract

Recurrent pregnancy loss (RPL) due to thyroid peroxidase antibody (TPOAb) syndrome remains a significant challenge in pregnancy. The current study offers better insights into miscarriages that occur due to the presence of TPOAb with euthyroid in pregnant women with a history of RPL. Out of the 347 women confirmed with unexplained RPL, only 70 (20.2%) tested positive for TPOAb (215±53). After eight women were excluded from the study due to failure to follow up, 62 participants (age range: 33±4.8 years; body mass index (BMI):25-30kg/m^2^ (58%) and >30kg/m^2^ (42%)) were included. The TPOAb-dependent RPL patients were divided according to their RPL types into 23 (30.7%) nulliparous (1˚) and 39 (69.3%) multiparous (2˚) patients, respectively. Out of the sample, 69.2% and 30.8% had a history of miscarriages during the 1^st^ and 2^nd^ trimesters, respectively. For treatment purposes, while screening for the TPOAb, the women received 50µg/day of L-thyroxine (LTx) for three months prior to pregnancy and during the first three months of pregnancy and were followed up until giving birth or miscarriage. Thyroxine treatment was correlated to successful normal births in 56.6% and 21.2% of pregnant women after 36 and during 28-36 weeks of gestation, respectively. However, miscarriages occurred in 18.1% and 4.1% of patients during 14-28 weeks and before 14 weeks of gestation, respectively. The current findings show the promising use of thyroxine in the control of RPL caused by euthyroid-based thyroid peroxidase antibody syndrome. This treatment has led to a significant number of women experiencing successful full-term pregnancies and giving birth to healthy babies.

## INTRODUCTION

A frequent consequence of pregnancy is a miscarriage, defined as the termination of a pregnancy before reaching 24 weeks of gestation. Miscarriage may affect as many as one out of every five women who try to conceive [1]. An estimated 6-10% of all pregnancies end in preterm birth, defined as the delivery of a baby before the 37^th^ week of gestation. Premature delivery, particularly before the 28^th^ week of pregnancy, is responsible for up to 85% of all newborn fatalities and 10% of surviving fetuses remain permanently disabled [2]. The annual cost of premature delivery in the United Kingdom alone is £93 million. Premature delivery and miscarriage are both believed to be associated with thyroid autoimmunity, according to research [3].

Thyroid autoantibodies seem to be prevalent in women of reproductive age. Women with a previous record of RPL have thyroid antibodies (TAb) occurring at rates between 17-33%. In contrast, for women diagnosed with subfertility, the rate is 10-31%, while the general population experiences an incidence rate of 6-20%. Hypothyroidism, caused by TAb, is a leading contributor to poor fertility in the industrialized world [1, 4]. Investigations have linked the existence of TAb, especially TPOAb, to miscarriage, premature delivery, and extreme neurocognitive consequences in newborns and even women with biochemically normal thyroid function (euthyroid) [5, 6]. Although two potential pathways have been hypothesized, the precise mechanism remains unclear. Initially, even in women with euthyroid, the occurrence of TAb may be linked to a slight decrease in the supply of thyroid hormones or a weakened potential of the thyroid gland to sufficiently fulfill the requirement for enhanced formation of thyroid hormones needed during pregnancy [7].

Research tried to determine whether levothyroxine treatment improves birth outcomes in women with euthyroid who screened positive for TAbs, since even slight deviations in thyroxine concentrations have been linked to unfavorable outcomes in pregnant women [8]. Furthermore, elevated levels of TAbs may reflect a more generalized autoimmune response in the body that may be harmful to the maturation of the placenta or the fetus [9].

Previous definitions of RPL showed a 1% prevalence in couples with three or more maternity losses. Further assessments have redefined RPL as more than one maternity loss in the last several years, which impacts less than five percent of couples. Proper management and therapy of RPL are direly needed [10]. Antibodies like TPOAb and TGAb are present in most women with thyroid autoimmunity, although TSH-receptor antibodies are uncommon. In nations with an adequate provision of iodine, 5.1% to 12.4% of pregnant women test positive for TPO-Ab [11, 12]. Thyroid autoimmunity, and TPOAb in particular, has an uncertain impact on RPL clinical prognosis. Furthermore, women of reproductive age are disproportionately impacted in the context of the broader RPL definition [13]. Thus, more details from clinical studies are needed to determine the real impact of TPOAb on the outcomes of pregnancy. Therefore, the current study was conducted to determine effective treatments to overcome miscarriages that occur due to the presence of TPOAb with euthyroid in pregnant women with a history of RPL.

## MATERIAL AND METHODS

### Study Population

The clinical trial included the recruitment of 347 women with unexplained RPL; however, only 70 (20.2%) participants tested positive for TPOAb (215±53), and eight women were excluded from the study due to failure to follow-up. As a result, 62 participants (age range: 33±4.8 years; body mass index (BMI):25-30kg/m^2^ (58%) and >30kg/m^2^ (42%)) were included.

The TPOAb-dependent RPL patients were divided according to their RPL types into 23 (30.7%) nulliparous (1°) and 39 (69.3%) multiparous (2°) patients, respectively. The blood levels were evaluated for follicle-stimulating hormone (FSH) and luteinizing hormone (LH) on the 2^nd^ day of the cycle, thyroid-stimulating hormone (TSH), T3, and T4 at any time, prolactin and Anti-Müllerian hormones regardless of menstrual cycles. TPOAb, and other antibodies for other autoimmune syndromes, such as antiphospholipid antibody syndrome, were examined in order to perform a diagnosis regarding the cause of RPL. These women had a history of miscarriages during the 1^st^ (69.2% of the sample) and 2^nd^ trimesters (30.8% of the sample). For treatment purposes, while screening for the TPOAb, the women received 50µg/day of LTx for three months prior to pregnancy and during the first three months of pregnancy. The pregnant women were followed up until giving birth or miscarriage. The trial lasted from May 2020 to March 2022.

The women who refused to participate or did not come back for follow-up visits were excluded from the sample. Furthermore, participants with self-reported pancreatitis, recent or major abdominal surgery, polycystic ovarian syndrome, or irregular menstruation were also excluded from the analysis.

### Statistical analysis

Prism v9 was used to analyze and graph data, in which the Mean and standard error of the mean (SEM) were followed. Results were considered significant at a p-value of less than 5%.

## RESULTS

**Figure 1:**
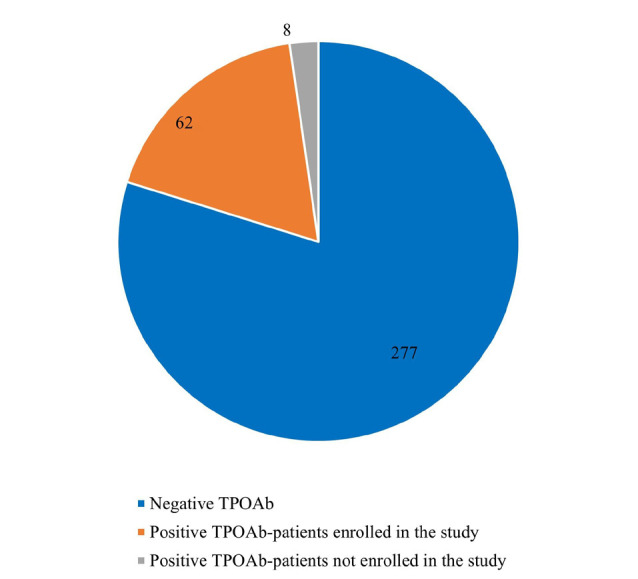
Patient data (patients tested negative for TPOAb, patients included in the treatment trial, and patients who dropped out of the trial)

**Figure 2:**
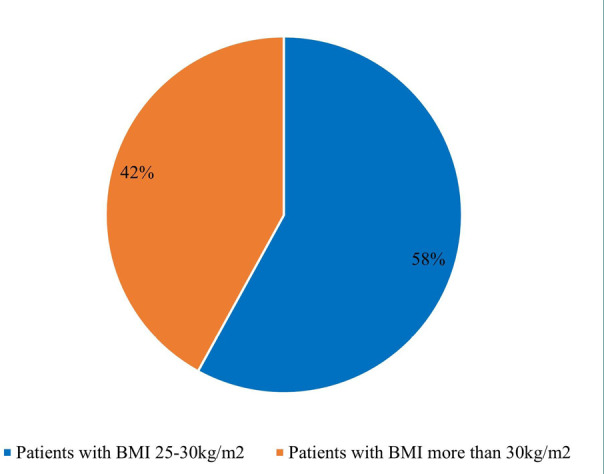
Patient data (%) (Patients with BMI 25-30kg/m2, and patients with BMI >30kg/m2)

Out of the 347 women with unexplained RPL in the initial sample, only 70 (20.2%) tested positive for TPOAb (215±53), and eight were excluded due to failure to follow up, leading to a final sample of 62 participants (age range: 33±4.8 years, body mass index (BMI): 25-30kg/m^2^ (58%) and >30kg/m^2^ (42%) (Figure 1, Figure 2).

**Table 1:** Gestation outcomes in women with thyroid peroxidase antibody-based recurrent pregnancy loss after thyroxine treatment for three months before and after conceiving

Gestational outcome	Percentage (%)
Normal births after 36 weeks	56.6
Normal births during 28-36 weeks	21.2
Miscarriages during 14-28 weeks	18.1
Miscarriages before 14 weeks	4.1
History of miscarriage	
Nulliparous women (before treatment)	30.8
Multiparous women (before treatment)	69.2

The pregnancy outcome revealed that the response to the thyroxine treatment showed successful (p<0.0001) normal births in 56.6% and 21.2% of pregnant women after 36 and at 28-36 weeks of gestation, respectively. However, miscarriages occurred in 18.1% and 4.1% of patients during 14-28 weeks and before 14 weeks of gestation, respectively. These results showcased a decreased occurrence and were highly significant (p<0.0001) (Figure 1).

## DISCUSSION

RPL, which refers to the loss of two or more pregnancies before the fetus reaches viability, represents a significant clinical concern. Multiple autoimmune disorders are among the primary etiologies of the RPL [14]. Women who had repeated miscarriages had greater levels of circulating anticoagulant than normal controls, as reported for the first time during the 1970s [15]. A further investigation revealed that these anticoagulants were lupus anticoagulant antibodies. A number of investigations since then have confirmed the association between autoimmune illnesses and miscarriage, including antiphospholipid syndrome and systemic lupus erythematous [16].

Thyroid autoantibodies have been linked to multiple miscarriages. It has been hypothesized that uncontrolled immunological activity at the fetal-maternal junction may result from the existence of thyroid autoantibodies. Women who have had several miscarriages were reported to have greater levels of circulating anti-thyroid antibodies than control subjects [17].

The purpose of this research was to determine if treating euthyroid women with TPOAb who have had a history of RPL with LTx is beneficial. Therapy of pregnant women is supported by data showing an increased risk of miscarriage for those with TSH levels over 2.5mIU/L [18]. Nevertheless, it remains debatable when exactly the need for LTx rises throughout pregnancy. In reality, once a woman decides to conceive a child, moving from a replacement to a partly suppressed LTx strategy resulted in appropriate blood free-thyroxine levels up until the initial post-conception hormonal assessment. However, the root cause of hypothyroidism seems to be the most important factor in deciding when and how much TH needs to be adjusted [19, 20].

Depending on the underlying cause of hypothyroidism, women need careful observation of the thyroid function after pregnancy and preparatory modifications to their LTx dose. According to the guidelines of the American Thyroid Association [21], all currently LTx-treated hypothyroid women should aim to optimize their thyroid function before attempting to conceive. Clinical research suggests that aiming for a maternal serum TSH value of less than 2.5 mIU/L is a realistic target [22]. For substantially lessening the possibility of moderate hypothyroidism at the beginning of the gestation, TSH levels should ideally be below 1.5 mIU/L. Even euthyroid women with TPOAb who do not progress to hypothyroidism or subclinical hypothyroidism throughout gestation, have significant shifts in the need for TH synthesis and biological activity beginning in the first trimester. The present study used the LTx treatment three months before and after Human Chorionic Gonadotroph (hCG) confirmation of conceiving, as supported by previous studies [23].

Having a sufficient amount of thyroid reserve is therefore essential for healthy implantation and subsequent pregnancy. Twenty-five percent of pregnancies terminate prematurely for unknown reasons, and this may be related to a failure of the body's endocrine system to adjust physiologically after implantation. In this study, it was observed that administering LTx to TPOAb euthyroid women with previous RPL reduced the risk of a subsequent miscarriage. In euthyroid women with TPOAb, treatment with LTx significantly reduced the risk of miscarriage and premature delivery. Pregnant women with or without TPOAb should be treated with LTx throughout gestation, according to recommendations from the Endocrine Society Guidelines to reduce the risk of obstetric problems [24].

The findings match and extend those of Rotondi *et al*. [19], who found that a preconception adjustment of LTx might lead to sufficient gestational thyroid function through the initial post-conception examination. The present study results support those by Dal Lago [22], who found that the use of LTx, three months before and after conceiving, in euthyroid women with TPOAb increased the percentage of normal births after a full-term of gestation in 59% and lowered miscarriages in 12% of treated women.

## CONCLUSION

The current findings show promising efficacy of thyroxine in the control of recurrent pregnancy loss caused by euthyroid-based thyroid peroxidase antibody syndrome. This treatment has resulted in a significant number of women achieving successful full-term pregnancies and normal births.

## Data Availability

Further data is available upon request.

## Personal thanks

I would like to express my sincere gratitude to all the individuals and organizations who have contributed to the publication of this research paper.
